# Zirconia CAD-CAM Crowns Behavior after Intraoral Digital Impression in Normal versus Dysfunctional Patients: 3 Years Retrospective Study

**DOI:** 10.1055/s-0043-1777350

**Published:** 2024-02-08

**Authors:** Francesco Ferrini, Francesco Gianfreda, Francesco Bova, Francesca Cattoni, Patrizio Bollero, Enrico Gherlone, Filiberto Mastrangelo

**Affiliations:** 1 Department of Dentistry, IRCCS Ospedale San Raffaele, Milan, Italy; 2 Department of System Medicine University of Rome “Tor Vergata”, Rome, Italy; 3 Centro di igiene orale e prevenzione, Department of Dentistry, IRCCS Ospedale San Raffaele, Milan, Italy; 4 Clinical and Experimental Medicine Department, University of Foggia, Foggia, Italy

**Keywords:** digital CAD-CAM, zirconia crowns, marginal adaptation, dental prosthesis, parafunctions

## Abstract

**Objectives** The aim of this study was to evaluate the clinical performance and possible complications of single zirconia crowns fabricated using an intraoral digital computer-aided design-computer-aided manufacturing (CAD-CAM) protocol in normal and dysfunctional patients after 3 years of follow-up.

**Materials and Methods** Seventy patients were included in this study. The teeth were prepared with a knife-edge marginal design, and temporary crowns were placed. Digital impressions were taken using optical scanning, and the frameworks were milled using the same technology. The veneering process was performed by the same dental technician. The occlusal corrections were made before cementation. The outcomes were evaluated in terms of survival, failures, and complications. The marginal adaptation of the crowns was also assessed.

**Results** The digital protocol for single zirconia crowns resulted in satisfactory outcomes, with high rates of survival and minimal complications after 3 years of follow-up. The marginal adaptation of the crowns was excellent, with 93% of the restorations achieving the ideal marginal adaptation, while 7% had minor deviations. Parafunctions were found in 41.9% of the prosthetic rehabilitation, but no significant differences were observed between the normal and dysfunctional groups regarding the survival and complications of the crowns.

**Conclusion** The digital protocol for single zirconia crowns is a reliable and predictable treatment option, even for patients with parafunction, when proper occlusal corrections are performed before cementation. The use of intraoral digital CAD-CAM technologies with optical impressions can simplify procedures, reduce the workflow time, and minimize the variables linked to the human factor.

## Introduction

In modern dentistry, the use of advanced technologies and materials is aimed at restoring tooth function and aesthetics while minimizing patient discomfort. 1 Classical impression techniques in combination with plaster master casts and porcelain-fused-metal (PFM) crowns have long been the gold standard in the manufacturing process for fixed implant-supported reconstructions. 2 3 4 However, conventional techniques have several associated drawbacks, including time-consuming and complex manufacturing steps with expensive manpower and equipment, a long list of materials with inconsistent quality, and interference with treatment steps during impression taking due to suffocation hazard, gagging, and taste irritation. 5

To overcome these challenges and improve outcomes, intraoral digital computer-aided design-computer-aided manufacturing (CAD-CAM) technologies are increasingly being used to simplify procedures, improve patient compliance, reduce workflow time, and minimize the variables linked to the human factor. 6 Modern CAD-CAM subtractive systems, in combination with light or laser digital intraoral impression scanners and graphics software, are being used to produce zirconia crowns, which are becoming increasingly popular in prostheses, especially when the interarch space is inadequate. 7

Despite the advantages of digital protocols for zirconia crown manufacturing, clinical practice has reported more crown fractures, chipping, and various complications. The literature suggests several potential reasons for these failures, including the quality of the zirconia-ceramic adhesion, specific crown defects, cementation defects, and higher occlusal module or parafunctions. 8

In particular, parafunctions in dentistry are defined as habitual or involuntary behaviors that can cause damage to the teeth, jaw joints, or muscles.

Parafunctions in dentistry are identified as habitual or involuntary behaviors capable of causing damage to the teeth, jaw joints, or muscles. These habits include bruxism (grinding and clenching), nail biting, chewing on objects, among other oral habits, 9 which could lead to dental issues such as tooth wear, temporomandibular joint disorders, and notably, fractures of prosthetic rehabilitations.

A systematic review by Leitão et al 10 encompassed 594 participants and 1657 single-tooth restorations, revealing that marginal integrity exhibited high success rate values across observation periods, with a notable exception in a subgroup of patients with bruxism, where a survival rate of 31.60% was recorded. This indicates that bruxism significantly influences the durability and integrity of zirconia restorations.

On the other hand, Tartaglia et al 11 evaluated the 7-year clinical outcomes of 303 zirconia core restorations in a general dental private practice, documenting an overall 7-year survival probability estimate of 0.966 for failures, with a cumulative survival rate of 94.7%. However, 16 restorations/abutment teeth (5%) encountered some complications including porcelain veneer fractures, which can be ascribed to various factors including parafunctional habits.

Therefore, it is imperative for dental professionals to evaluate patients for parafunctional habits, offering suitable treatment or preventive measures such as the utilization of night guards or other oral appliances to protect both teeth and restorations. 12

While digital CAD-CAM technologies burgeon with potential to streamline procedures, foster patient compliance, and reduce workflow time, their application necessitates thorough training and expertise.

The aim of this study was to evaluate with a 3 years follow-up, the possible relationship between length, survival, failures, and complications of single zirconia crowns made with digital protocol in normal and dysfunctional patients.

## Materials and Methods

### Study Design and Participants

This study utilized a retrospective design and included 70 patients who required dental crown restorations at the Dental Clinic of Vita Salute University San Raffaele Milan-Italy between January 2011 and December 2014.

Informed consent was obtained from all the study participants before the prosthetic treatment.

The study was conducted in accordance with the principles of the Declaration of Helsinki.

Inclusion criteria were age between 18 and 65 years, individuals without extensive prosthetic rehabilitations of old date exceeding 3 elements per arch, individuals without devitalized teeth unprotected by prosthetic elements, individuals who are not pregnant, and individuals who are not in treatment with bite.

Data was extracted from the clinic's electronic medical records, including demographic information, rehabilitation characteristics, and postoperative outcomes. Patients who required dental crown restorations were included in the study. Patients with a history of systemic disease or other oral conditions that could affect crown restoration were excluded from the study.

### Digital Impression and Crown Restoration Procedure

**Fig. 1:**
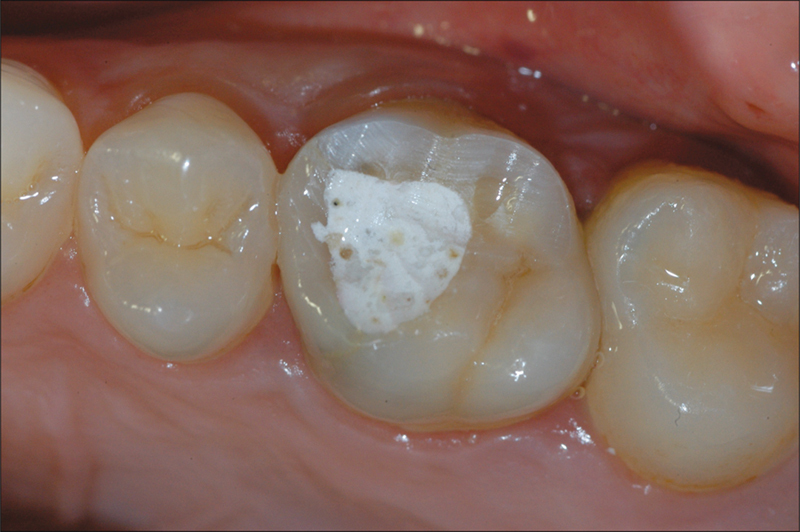
Element structurally compromised and treated endodontically. Prosthetic rehabilitation with crown is required.

**Fig. 2:**
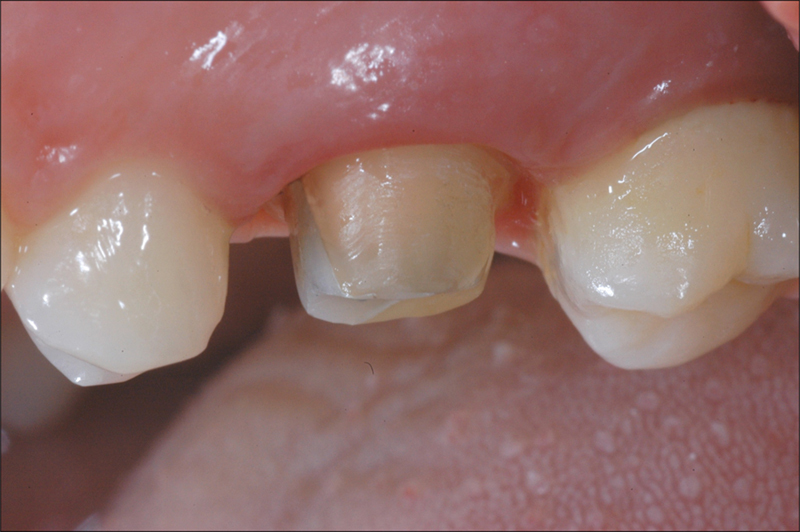
Element prepared with a knife-edge finishing line. Periodontal tissues conditioned by the provisional during optical impression taking.

Tooth preparation was performed with a knife-edge marginal design, and provisional composite-resin crowns were placed on the same day. After 8 weeks of tissue conditioning, the patients underwent a digital impression using Lava™ Powder for Chair-side Oral Scanner (3M ESPE, St. Paul, Minnesota, United States), which involved applying a thin dust layer on the teeth, as recommended by the manufacturer ( Figs. 1 and 2 ). To achieve adequate gingival retraction, two retraction cords of varying diameters were gently placed into the gingival sulcus, with the narrower cord (000-Retraction cord, Ultradent Products, South Jordan, Utah, United States) positioned apically to the wider cord (0-Retraction cord, Ultradent Products).

The gingival margin, without powder excesses and possible elements of distortion like bubbles or undefined areas, was evaluated with the digital impression image screen magnified on the PC screen. Adjacent teeth were evaluated as well. If any problem was found, the scanning process was repeated.

The workflow required a second scan for the opposite arch and a third scan for bite registration (Lava Chairside Oral Scanner, 3M ESPE, St. Paul, Minnesota, United States).

After the digital scan was accepted, all the frameworks were designed and milled by the same center using 3M ESPE Lava Form CNC Mill system (Lava, 3M ESPE).

The veneering process was made using a leucite-based material (veneering ceramic was leucite-based (Creation Zi-CT, Willi Geller, Meiningen, Austria) and performed by the same dental technician. The occlusal corrections were made by means of a diamond bur before the ceramic glazing procedure.

**Fig. 3:**
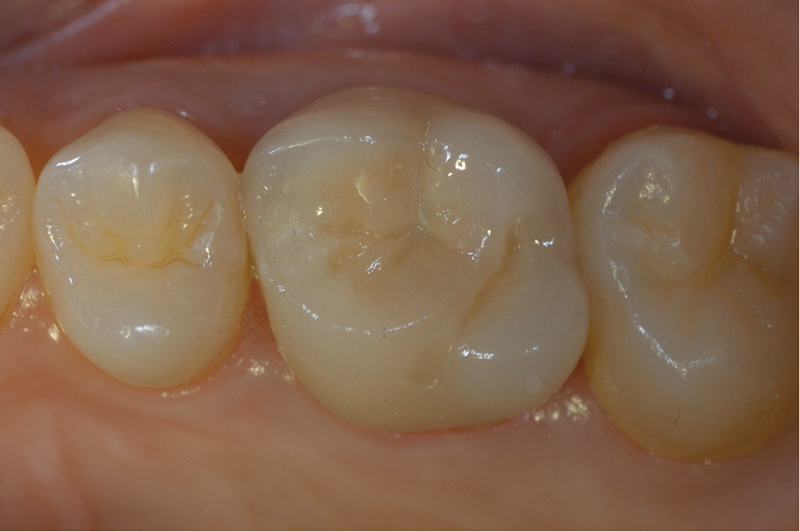
Definitive crown delivery.

Before cementation, all abutments were carefully cleaned with a 70% alcoholic solution and air-dried. Cementation was performed using dual-curing, self-adhesive resin cement (Relyx, Unicem - 3M ESPE; Figs. 3 4 5 ).

**Fig. 4:**
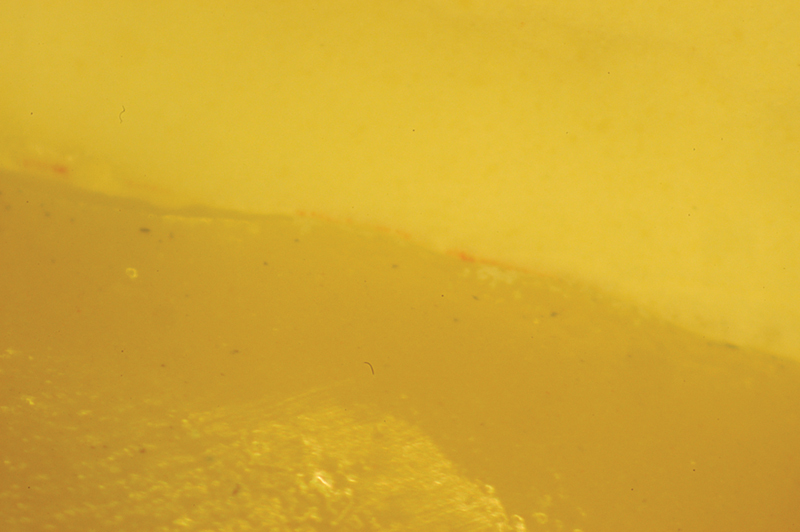
Evaluation of the precision of the finishing margin on the model at 40x magnification under an optical microscope.

**Fig. 5:**
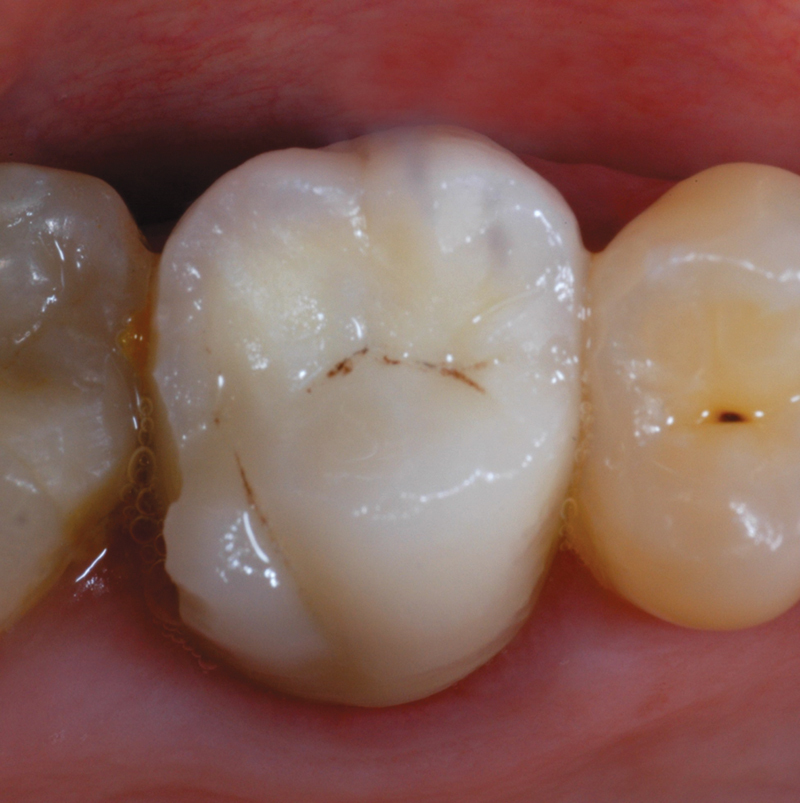
Chipping of the distal–palatal cusp of a tooth 1.6.

### Data Collection

A specific data-form was used to record the following information for each patient: gender, age, parafunction, marginal adaptation, and position of the crowns as well as the day of the restoration delivery. After anamnestic and clinical objective evaluation, the patients were selected, as parafunctional, if they scored higher than 2 with the Smith and Knight's index 13 and no signs of erosion, in addition to a self-reported questionnaire. Marginal adaptation was evaluated by means of a blunt explorer accordingly with FDI World Dental Federation (FDI) clinical recommendations 14 and the following codes were used:

A= no clinically detectable gap. Margins represent a harmonious continuation of the outline at the tooth/restoration transition.

B= marginal integrity deviates from the ideal, but could be upgraded to ideal by polishing. Small marginal chip fracture of the restoration can be eliminated by polishing and/or a localized gap was just perceptible with a dental probe more than 50 µm and less than 150 µm;

C= leakage/discoloration was present but limited to the border area of the margins. Generalized marginal gap more than 150 µm, but less than 250 µm is easily perceptible on probing but cannot be modified without minor damage to the tooth or surrounding tissue, and was not considered to result in long-term negative consequences for the tooth or surrounding tissue if left untreated. Presence of several small marginal fractures that were unlikely to causes long-term effects.

D= localized gap larger than 250 µm may result in exposure of dentin or base. Repair was necessary for prophylactic reasons.

E= generalized gap larger than 250 µm or the restoration was loose but *in situ* replacement was necessary to prevent further damage or there were large fractures at the margins and loss of material was too extensive to be repaired.

After 12, 24, and 36 months, the patients were re-evaluated adding data regarding crown chipping and fractures (yes/no). Crown failure was defined as restorations having been removed, and crown complication was considered as one or more events affecting function and/or esthetics.

For complications, additional data regarding the chipping/fracture location were recorded.

### Statistical Analysis

The statistical analysis was made by a binary logistic regression analysis, which is a type of regression analysis used to model the relationship between a binary dependent variable and one or more independent variables. SPSS (Statistical Package for the Social Sciences, SPSS Inc., Chicago, Illinois, United States) was used as statistical software. SPSS is particularly well-suited for analyzing and visualizing complex datasets, making it a valuable tool for this kind of studies.

## Results

**Fig. 6:**
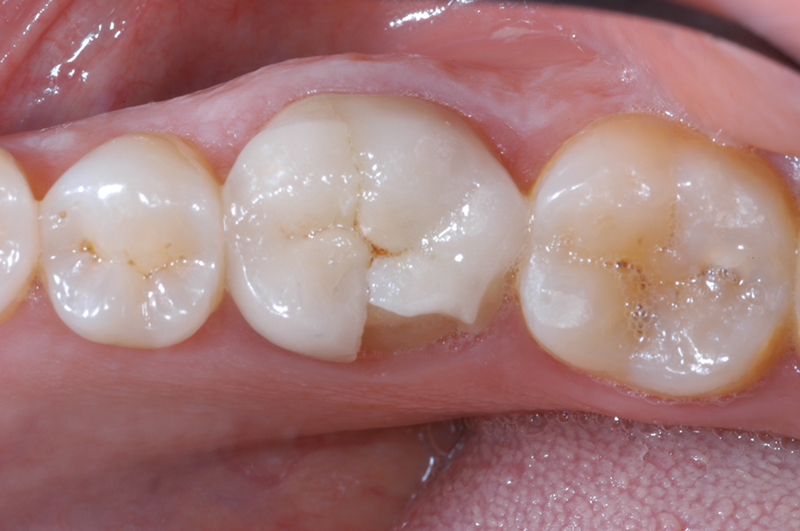
Fracture of the distal–lingual shear cusp of a tooth 4.6.

**Fig. 7:**
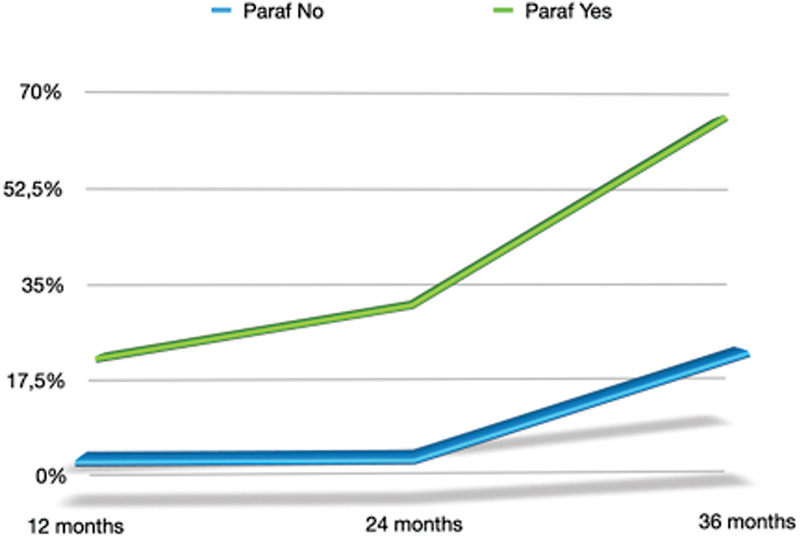
Distribution of complications in parafunctional and nonparafunctional patients.

The study included 70 patients, comprising 39 males and 31 females, with a mean age of 45.9 years (range 24–75 years; standard deviation [SD] = 11.6; Table 1 ). Eighty-six single crowns were placed in these patients, with 13 crowns inserted in the anterior area (9 incisors, 4 canines) and 73 in posterior sites (27 premolars, 46 molars). Of these, 36 single crowns were placed in parafunctional patients (41.9%) and 50 single crowns were cemented in normal patients (58.1%; Tables 2 and 3 ). At baseline, minor occlusal adjustments were needed in 11.6% of the crowns. No clinical detectable gaps were shown in 77.9% (A adaptation) and 22.1% of the patients showed B adaptation. No evidence of C, D, or E adaptation was found. After 12 months of follow-up, eight zirconia crowns showed chipping (5 parafunctional patients—3 normal patients), and two patients (1 parafunctional patients–1 normal patients) showed hypersensitivity, which disappeared at the following recalls. At the same time of follow-up, it was observed that there was no secondary decay, loss of tooth vitality, gingival recession or tooth extraction for periodontal or endodontic reasons. After 24 months of follow-up, only two crowns showed chipping, all in parafunctional patients. At the same time, there was no evidence of further complications. At 36 months of the following recalls, 14 zirconia crowns showed chipping (7 parafunctional patients–7 normal patients), and there was no evidence of further complications. In total, after the 36 months follow-up, it was possible to observe that 24 crowns chipped (27.9%) and two crowns failed (2.3%; Figs. 5 and 6 ). Crowns placed in parafunctional patients chipped more than in normal patients (odds ratio [OR] = 2.54; 95% confidence interval [CI]: 0.97–6.67; *p* = 0.575). Crowns that needed occlusal adjustments chipped more than crowns with a perfect occlusion (OR = 3.00; 95% CI: 0.78–11.50; *p* = 0.109). Crowns with B adaptation chipped more than crowns with A adaptation (OR = 5.71; 95% CI: 1.91–17.05; *p* = 0.002; Table 4 ). A second level of data analysis was conducted to investigate if the parafunction conditions showed a specific correlation between complications and the occlusal adjustments or adaptation score. Crowns that needed occlusal adjustments in parafunctional patients displayed more chippings than crowns that needed occlusal adjustments in normal patients (OR = 7.86; 95% CI: 0.28–217.12; *p* = 0.223). Crowns with B adaptation in parafunctional patients had more chippings than crowns with B adaptation in normal patients (OR = 5.25; 95% CI: 0.70–39.48; *p* = 0.107; Table 5 , Fig. 7 ).

**Table 1:** Age distribution of patients treated

Tooth position	# of teeth
Incisor	9
Canine	4
Premolar	27
Molar	46
Total	86

**Table 2:** Tooth requiring prosthetic rehabilitation with crown

Age group	
20–29	6
30–39	26
40–49	21
50–59	22
60–69	5
70–79	6
Grand total	86

**Table 3:** Distribution of parafunctions

Parafunctions	12 m	12 m %	24 m	24 m %	36 m	36 m %	Total	Total %
paraf NO	3	8%	0	0%	7	18%	10	25%
paraf YES	5	23%	2	9%	7	32%	14	64%
Total	8		2		14		24	

**Table 4:** Binary regression of parafunctions versus normal patients, occlusal adjustments versus perfect occlusion and adaptation B versus A

	OR	95% CI	*p* -Value
Parafunctions vs. normal	2.54	0.97–6.67	0.575
Occlusal adjustments vs. perfect occlusion	3	0.78–11.50	0.109
Adaptation B vs. A	5.71	1.91–17.05	0.002

Abbreviations: CI, confidence interval; OR, odds ratio.

**Table 5:** Binary regression in parafunctional and nonparafunctional patients

	OR	95% CI	*p* -Value
Occlusal adj. parafunctional patients vs. occlusal adj. NOT parafunctional patients	7.86	0.28–217.12	0.223
Adaptation B parafunctional patients vs. adaptation B NOT parafunctional patients	5.25	0.70–39.48	0.107

Abbreviations: CI, confidence interval; OR, odds ratio.

## Discussion

The primary objective of this investigation was to appraise the clinical efficacy of single zirconia crowns in both standard and parafunctional patients. The evidence unearthed a higher propensity for chipping in parafunctional patients, albeit statistically nonsignificant, underscoring the necessity for an in-depth exploration into the occlusal dynamics, especially in individuals exhibiting parafunctional behaviors. The results showed that 86 single crowns were placed in 70 patients, comprising 39 males and 31 females, with a mean age of 45.9 years (range 24–75 years; SD = 11.6). Of these, 36 single crowns were placed in parafunctional patients (41.9%) and 50 single crowns were cemented in normal patients (58.1%). Most of the crowns were placed in the posterior region (27 premolars, 46 molars), and only 13 crowns were placed in the anterior area (9 incisors, 4 canines). At baseline, 11.6% of the crowns required minor occlusal adjustments. After 12 months of follow-up, eight zirconia crowns showed chipping, and two patients experienced hypersensitivity, which disappeared in the subsequent recalls. At 24 months, only two crowns showed chipping, both in parafunctional patients. At 36 months, 14 zirconia crowns showed chipping, and two crowns failed. The overall chipping rate after 36 months was 27.9%, and the failure rate was 2.3%. The study found that parafunctional patients had a higher chipping rate than normal patients, although the difference was not statistically significant. Crowns that required occlusal adjustments and had B adaptation showed a statistically significant association with chipping. However, the study did not find any evidence of secondary decay, loss of tooth vitality, gingival recession, or tooth extraction for periodontal or endodontic reasons. The results of this study are consistent with previous research in the literature that has evaluated the clinical performance of zirconia single crowns. For example, a retrospective study conducted by Tanner et al 15 found that the overall survival rate of zirconia single crowns was 94.2% after a mean follow-up of 5.7 years. The most common complication was chipping, which occurred in 6.2% of the cases. Another systematic review and meta-analysis by Sailer et al 16 evaluated the clinical performance of zirconia single crowns and reported a 5-year survival rate of 96.5%. The most common complication was also chipping, which occurred in 3.9% of the cases. In addition, the review found that the survival rate of zirconia single crowns was comparable to that of metal-ceramic crowns. In conclusion, the results of this study suggest that zirconia single crowns can provide satisfactory clinical performance in both normal and parafunctional patients. However, clinicians should pay attention to occlusal adjustments and adaptation scores to minimize the risk of chipping.

More than two-thirds (69,2%) of the complications in this study occurred on the buccal cusps of maxillary teeth and the lingual cusps of mandibular teeth. These cusps have a chance to contact the opposite dentition during lateral movements rather than centric occlusion. For this reason, it is conceivable that improving occlusal anatomy, especially in parafunctional patients, should reduce the complication rate. In addition, the most common complication was chipping, which occurred in 27.9% of the crowns, particularly in parafunctional patients and those with occlusal adjustments or poor adaptation scores.

Dysfunctional and parafunctional patients often present unique challenges in restorative dentistry. Parafunctional habits, such as bruxism (teeth grinding and clenching), can significantly impact the longevity and performance of dental restorations including zirconia crowns. Zirconia, known for its excellent mechanical properties, biocompatibility, and aesthetics, has become a popular choice for crown restorations over the last decade. 17 However, the management of patients with severely worn dentition due to parafunctional habits like bruxism is challenging, especially when there's a loss of occlusal vertical dimension and tooth structure. 18

Clinical evaluations of zirconia-based restorations show promising results, with mechanical failures primarily observed in patients exhibiting parafunctional habits over a period of up to 5 years. 19 Bruxism, a common parafunctional habit, occurs both during sleep and wakefulness, often leading to various complications like chipping and fractures in restorative materials including zirconia. 20

The robustness of zirconia crowns in withstanding the occlusal forces generated by parafunctional habits is a topic of clinical interest. Some studies suggest that while zirconia crowns exhibit high fracture resistance, the occurrence of chipping, particularly in the occlusal and incisal areas, is a concern in parafunctional patients. The demand for occlusal adjustments in these patients could potentially predispose zirconia crowns to chipping or fracture. Hence, addressing the occlusal anatomy, especially in parafunctional patients, is crucial to reducing the complication rate.

Moreover, the interim management during the phase of complete oral rehabilitation in dysfunctional and parafunctional patients is often complex due to the associated occlusal and structural challenges. The use of zirconia or other materials in such rehabilitations should be well-thought-out, with a thorough understanding of the patient's occlusal dynamics and parafunctional habits.

Future studies should aim at evaluating the long-term performance of zirconia crowns in a cohort of patients with defined parafunctional habits. Randomized controlled trials comparing zirconia with other restorative materials in dysfunctional and parafunctional patients could provide valuable insights into the material's performance and durability in such challenging clinical scenarios. Moreover, studies targeting the optimization of occlusal adjustments and the management of parafunctional habits could contribute to enhancing the clinical success of zirconia crowns in these patient populations.

Furthermore, in this study the crowns-patient ratio confounding factor is reduced with a mean of 1,2 crowns for each patient, in contrast with other studies in with the ratio was more than three. 21 22 23 24

One of the limitations of this study are the impossibility of being able to carry out a classification of the parafunctional habits of the patients. However, it has been pointed out how difficult can be to grade parafunctional patients. 25 Potentially a future study should randomly evaluate a cohort of patients with the same types of parafunctions treated with different types of material for the fabrication of CAD-CAM monolithic crowns.

Zirconia crowns are durable but present some issues. Clinical evaluations have shown crown or antagonist tooth fractures and crown abrasion. 26 The hardness of zirconia may cause abrasive wear on opposing teeth. Monolithic zirconia crowns prevent veneer chipping seen in bilayer restorations and allow minimally invasive tooth preparations but may lack in aesthetic appeal compared with bilayer crowns. 27 Early complications include localized gingival irritation, postoperative tooth sensitivity, and pulp exposure during preparation. 28 Esthetic limitations like translucency and shade matching affect zirconia's use in anterior restorations. 29 Zirconia-based all-ceramic crowns for molar teeth with metal antagonist occlusion should be undertaken cautiously due to risks like veneering ceramic fracture. 30

The presented study divulges several limitations that could potentially impact the validity and generalizability of the findings. One paramount limitation highlighted is the inability to classify the parafunctional habits of the patients involved. Parafunctional habits, such as bruxism, significantly influence the durability and performance of dental restorations like zirconia crowns. The study suggests that a thorough classification of parafunctional habits could provide a more nuanced understanding of how different types of parafunctions interact with various restorative materials. Furthermore, the lack of a randomized controlled trial design is a notable limitation, as it hinders the establishment of causal relationships between the observed outcomes and the variables investigated. The study also mentions the crowns-patient ratio as a confounding factor, albeit it has attempted to mitigate this by maintaining a relatively low mean of 1.2 crowns per patient. Additionally, the absence of long-term data and a lack of comparison with other restorative materials might limit the comprehensiveness and the depth of the insights gained. Future investigations could benefit from addressing these limitations by employing a randomized controlled trial design, extending the follow-up period, and comparing the performance of zirconia crowns with other restorative materials in a well-defined cohort of patients with classified parafunctional habits.

## Conclusion

In conclusion, this retrospective clinical study evaluated the long-term clinical outcomes and complications of zirconia single crowns placed in both normal and parafunctional patients. After a 36-month follow-up period, the overall survival rate was 97.7%, with only two crowns failing. Despite these complications, no secondary decay, loss of tooth vitality, or gingival recession was observed, indicating the zirconia crowns to be a suitable restorative option for both normal and parafunctional patients. Overall, these findings suggest that careful consideration of patient factors, such as parafunctional habits and occlusal adjustments, can help to reduce the risk of complications and improve the long-term success of zirconia single crowns.

Certainly, further studies are needed to confirm the encouraging findings, through an increase of the test group of dysfunctional patients and a extending the follow-up period.
